# Rice Body Formation in an Enterococcus faecalis-Infected Total Hip Arthroplasty Causing Sciatic Nerve Compression

**DOI:** 10.7759/cureus.67798

**Published:** 2024-08-26

**Authors:** Van Jet Leong, Azlina A Abbas, Khairul A Ayob, Kwong Weng Loh, Nazarina A Rahman, Veenesh Selvaratnam

**Affiliations:** 1 National Orthopaedic Centre of Excellence for Research and Learning (NOCERAL) Department of Orthopaedic Surgery, Faculty of Medicine, Universiti Malaya, Kuala Lumpur, MYS; 2 Department of Pathology, Faculty of Medicine, Universiti Malaya, Kuala Lumpur, MYS

**Keywords:** cumars construct, hip swelling, periprosthetic joint infection, total hip arthroplasty, rice bodies

## Abstract

We present a case of a 73-year-old lady with a previous uncemented left total hip arthroplasty (THA) three years prior to her current presentation. She presented with an enlarging ‘granulomatous'-looking swelling at the distal aspect of her THA scar for three months that was associated with shooting pain from the posterior aspect of her hip radiating down to her foot. The culture and sensitivity of her hip aspirate revealed the growth of *Enterococcus faecalis*. She underwent revision surgery utilising a ‘well-fixed’ Exeter custom-made articulating spacer (CUMARS). Intra-operatively, a large encapsulated cyst containing rice bodies was discovered deep within the fascia lata. A complete excision of this cyst was performed. Post-operatively, the patient was treated with two weeks of IV antibiotics and ten weeks of oral antibiotics. Histopathological examination confirmed the presence of rice bodies, with no malignancy seen. We aim to highlight the possibility of rice body cyst formation in the setting of a periprosthetic joint infection (PJI) around a THA and the importance of early treatment in such cases. This is the first published report of a rice body cyst formation in an infected THA.

## Introduction

Following a total hip arthroplasty (THA), many complications may arise, necessitating a revision surgery. One of which is an infection of the prosthetic joint and its surrounding soft tissues [1]. Patients with a periprosthetic hip joint infection often present with a painful, erythematous, and warm swelling of the hip. However, there are many differentials for a patient with painful hip swelling post-THA. Apart from infection, haematoma, malignancy, wear debris, hip bursitis, and inflammation of the hip synovium are possibilities [2].

Periprosthetic joint infections (PJI) following a THA are not uncommon occurrences, given the increasing number of hip arthroplasties being performed. However, PJIs are considered one of the most feared and severe complications, as they have been linked with increased healthcare costs due to the necessity of revision surgeries, reduced quality of life, and increased risk of mortality [3]. The major approaches for the surgical management of PJI can be either debridement and implant retention (DAIR), single-stage or a two-stage implant exchange [4]. Our centre utilises a single-stage ‘well-fixed’ custom-made articulating spacer (CUMARS) construct with antibiotic-loaded bone cement to eradicate infection and restore anatomical joint function.

Though no formal association has been established, there have been increasing reports of rice body cyst formation in patients with previous orthopaedic implants [2,5,6]. The term rice bodies was first coined in 1895 by Riese when it was discovered in patients with tuberculous arthritis [7]. Macroscopically, they resemble grains of polished white rice, hence their given name. These rice bodies are usually formed as a response to conditions predisposing to chronic synovial inflammation, such as tuberculous arthritis and rheumatoid arthritis [8]. Though there is no significant association between the formation of rice bodies and the course of the underlying disease [9], the size of the rice body cysts may vary, leading to possible compression of their surrounding neurovascular and musculoskeletal structures [2]. Consequently, this gives rise to debilitating complications for patients, such as limitation of joint range of motion and neuropathic pain.

While the formation of rice body cysts is a rare occurrence, it should still be considered in an array of differential diagnoses in patients presenting with hip pain and swelling, especially in patients with a prior history of orthopaedic implants, such as a THA.

We aim to report a case of a patient who had an infected THA that was complicated with rice body cyst formation compressing the sciatic nerve. This case also demonstrates the rare association of rice body formation with an infected THA and the importance of timely intervention. To the best of our knowledge, this is the first described case of rice body formation in association with an infected THA in the literature.

## Case presentation

**Figure 1:**
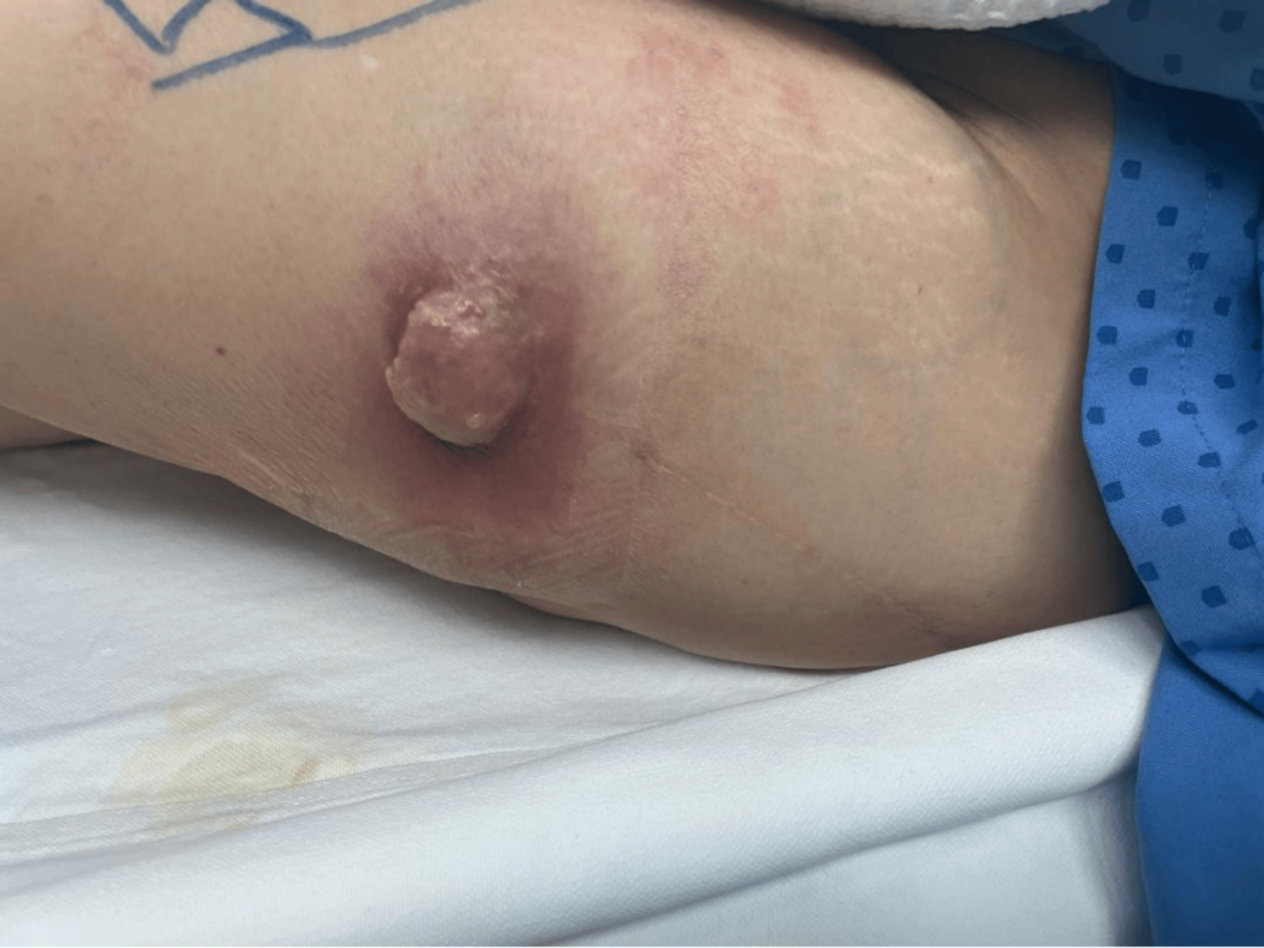
Left lateral thigh showing a ‘granulomatous’ like lesion just distal to the THA scar which was discharging serous fluid

This is the case of a 73-year-old Filipino lady with a background history of left hip osteoarthritis who was treated with an uncemented left THA three years prior to her current presentation. She presented with a ‘granulomatous'-looking swelling that was protruding over the lateral aspect of her left thigh (Figure 1), just distal to the previous left THA scar. The swelling had persisted for the last three months. The swelling was rapidly growing in size, starting from the size of a coin and eventually reaching the size of a ping-pong ball during her initial presentation. She reports that the swelling was erythematous, warm, and tender, with copious amounts of yellowish serous discharge that required daily dressing changes. In association with the swelling, she also experienced shooting pain from the posterior aspect of her left hip that radiated down to her foot. Her pain was suggestive of sciatic nerve compression. She was still able to ambulate and reported no restriction in the movement of her left hip. Of note, she did not have any chronic medical illnesses such as rheumatoid arthritis, systemic lupus erythematosus, or past tuberculous infections.

**Figure 2:**
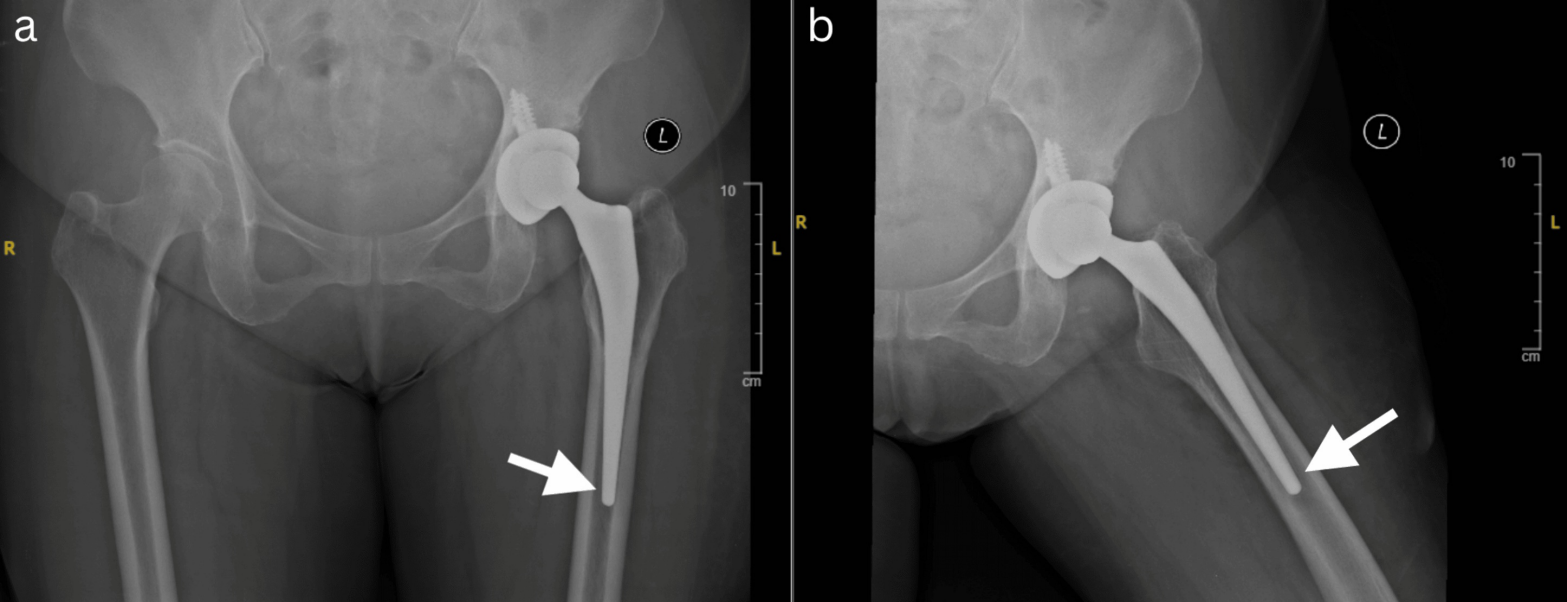
AP (a) and lateral (b) plain radiographs showing radiolucency at the distal stem (see arrow) of the THA

**Figure 3:**
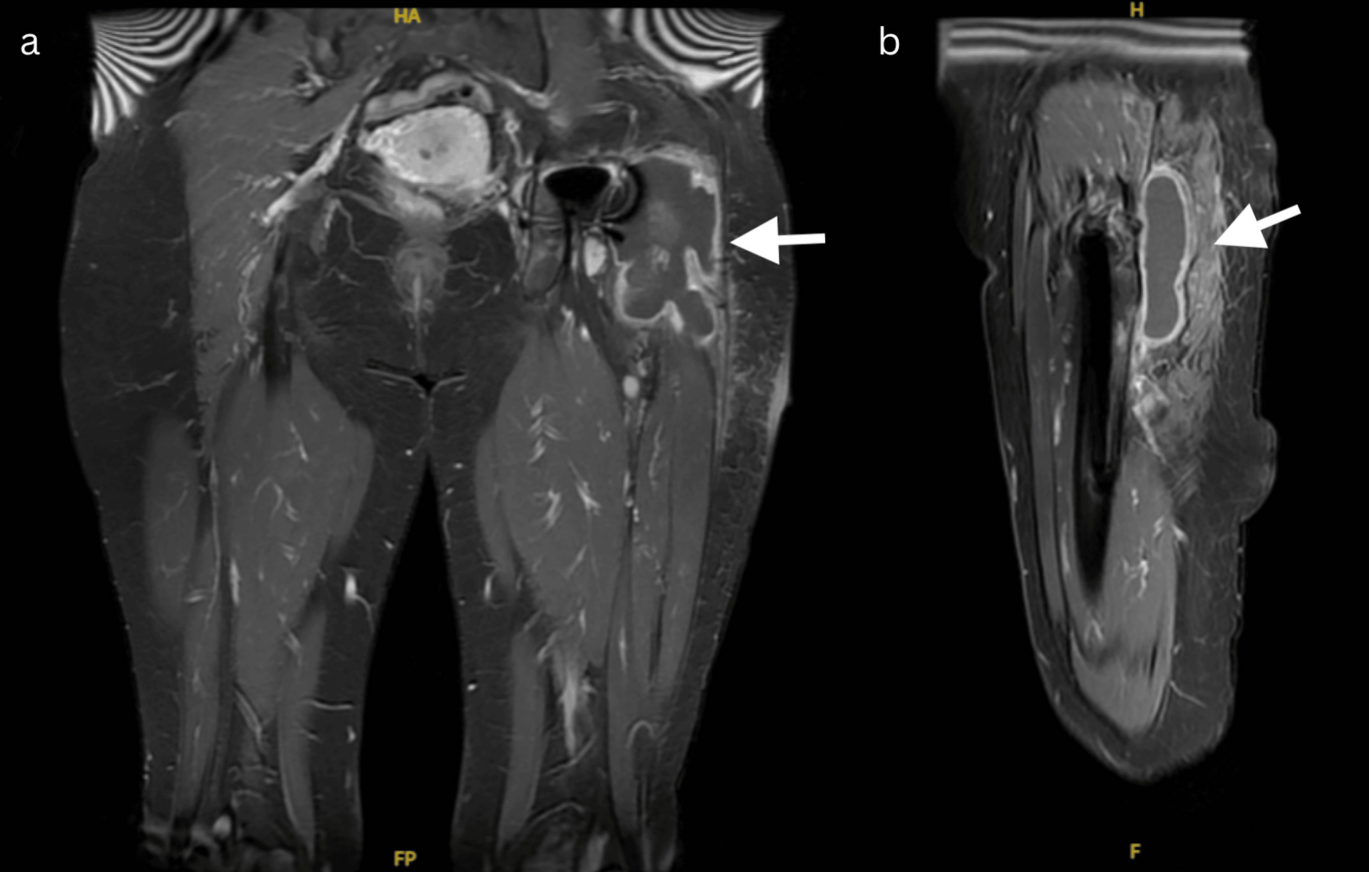
Magnetic resonance imaging showed a large complex fluid collection (3.9 cm × 7 cm × 9.6 cm) with a low signal on T1 in coronal (a) and sagittal view (b)

Laboratory investigations at the time of her presentation revealed a CRP of 29.2 mg/L (<5 mg/L) and an ESR of 71 mm/h (<29 mm/h). Plain radiographs of the hip showed radiolucency around the distal tip of the THA stem (Figure 2). Magnetic resonance imaging showed a large complex fluid collection (3.9 cm × 7 cm × 9.6 cm) with a low signal on T1 (Figure 3). There was also peripheral wall enhancement post-contrast. The collection extended posteriorly to the greater trochanter and deep to the iliotibial tract.

**Figure 4:**
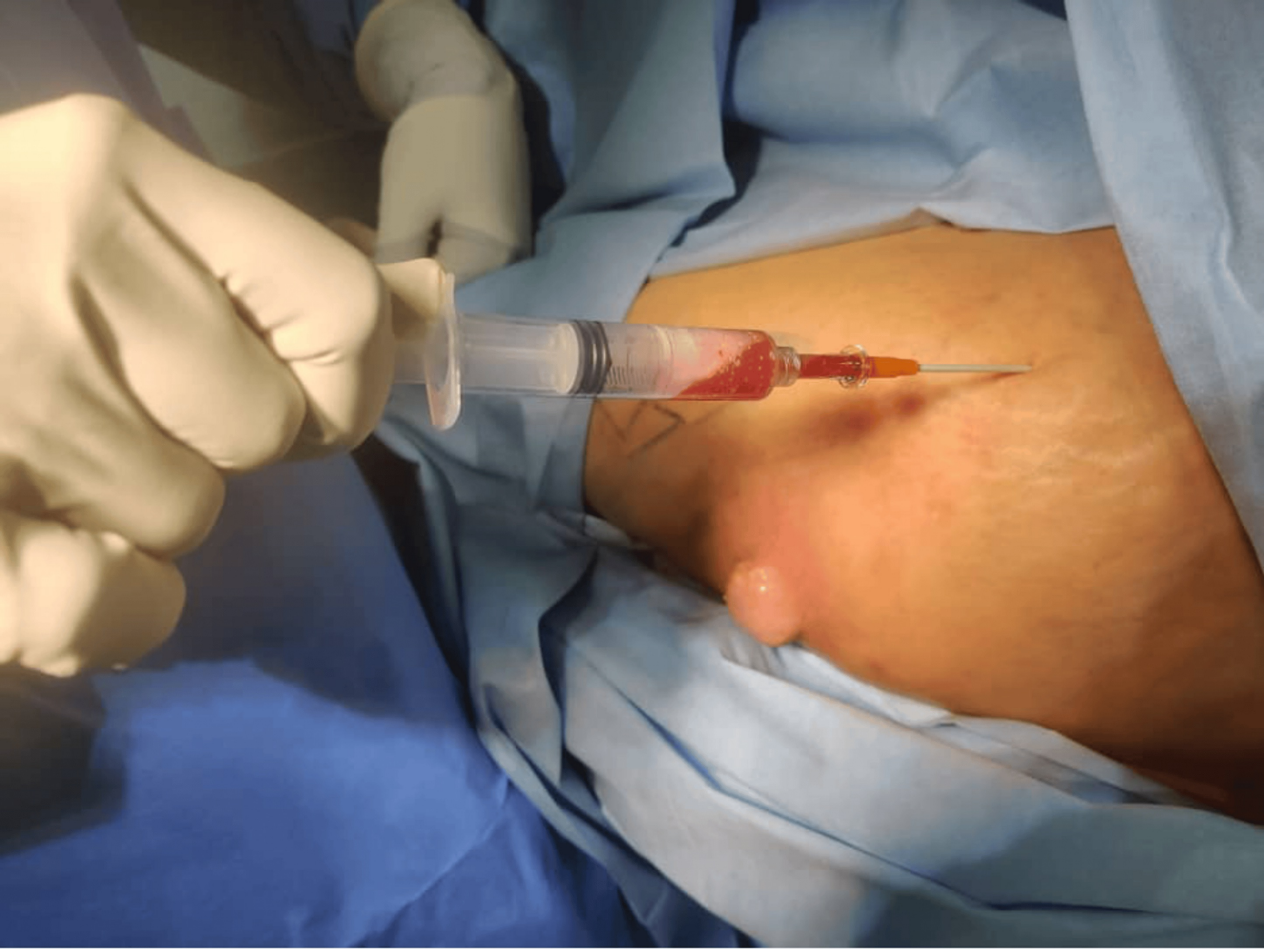
Aspiration of the left hip under aseptic condition yielding 3 ml of turbid with slightly blood stained fluid

Aspiration of this patient’s left hip under sterile conditions in the operating theatre with image intensifier (II) guidance was performed. It yielded 3 ml of turbid, slightly blood-stained fluid (Figure 4). The aspirate was sent for culture and grew Enterococcus faecalis, which was sensitive to ampicillin and benzylpenicillin. However, it is worth noting that the aspirate was negative for acid-fast bacilli.

**Figure 5:**
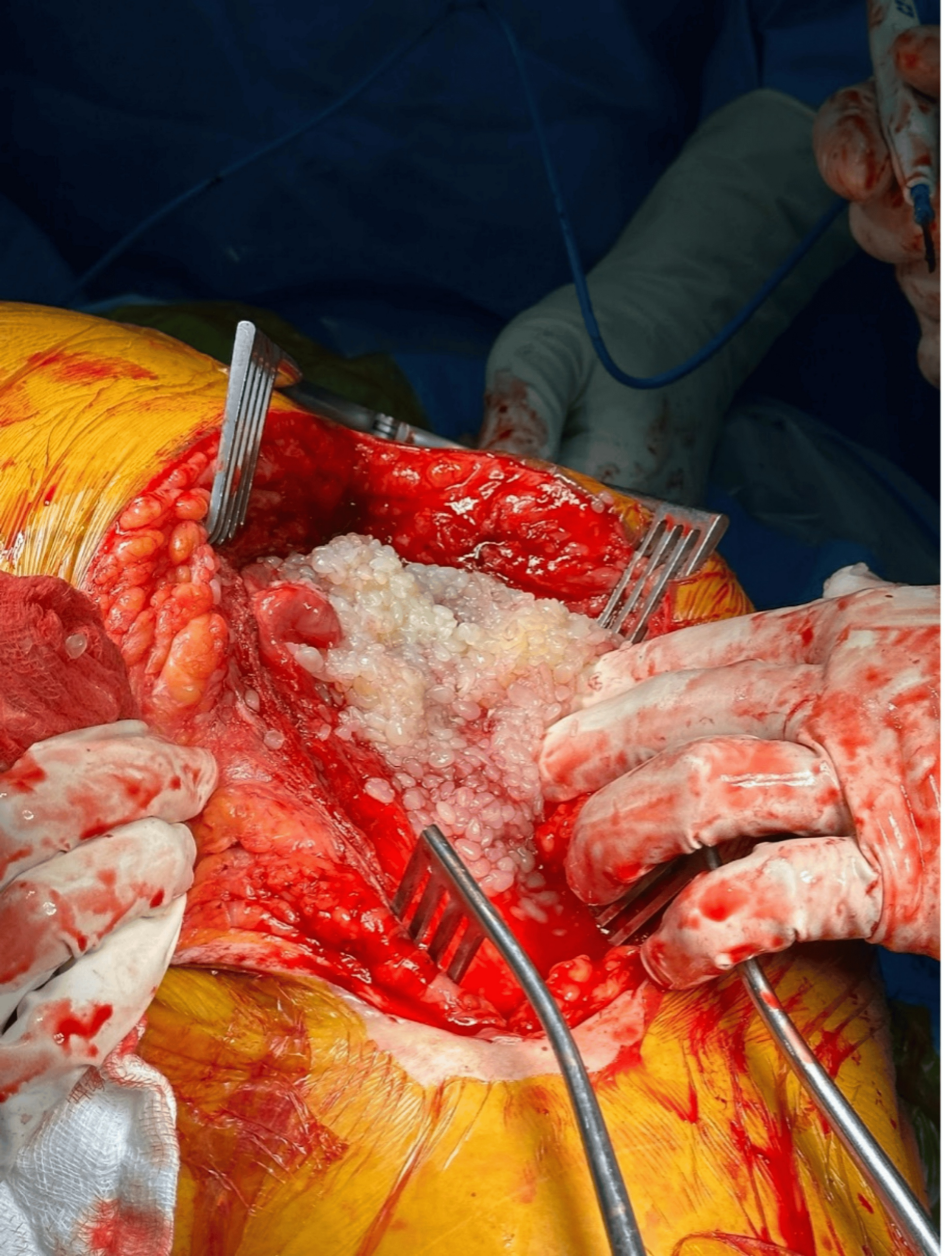
Multiple rice bodies just deep to the fascia lata which was compressing the sciatic nerve posteriorly

**Figure 6:**
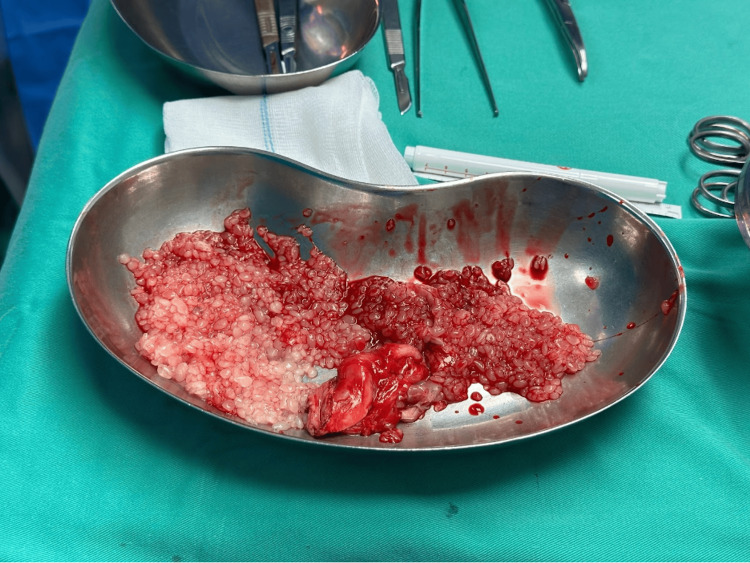
Multiple rice bodies which was sent off for microbiology and histological studies

As the aspirate confirmed the presence of an infection, this patient underwent revision THA with a ‘well fixed’ CUMARS construct [10]. Intraoperatively, the old scar was excised together with the external swelling. The posterior approach was utilised as this was the previously used approach. Deep to the fascia, we encountered a 10 cm by 8 cm cyst containing numerous loose structures that had the resemblance of polished rice grains (Figure 5), known as “rice bodies” in the literature [2,5,6], which were posterior lateral to the greater trochanter. The rice bodies were highly organised in a cyst, which was excised completely and sent for histopathological examination (Figure 6). This cyst containing rice bodies was compressing the sciatic nerve. The sciatic nerve was explored and was normal in appearance and continuity.

**Figure 7:**
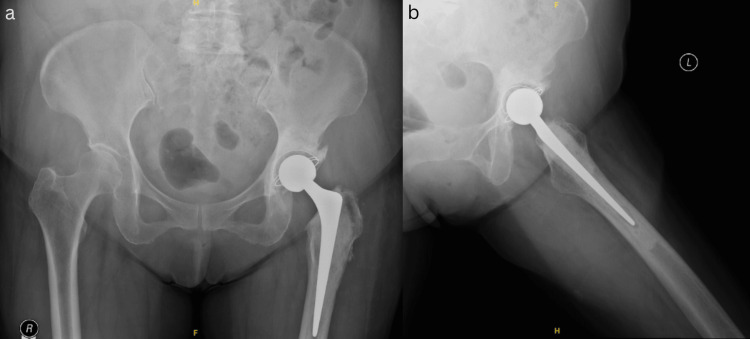
Post-operative X-rays of the pelvis in AP view (a) and left hip in lateral view (b)

Tissue samples from the hip acetabulum and femur were also sent for mycobacteriology diagnostics to look for a causative organism. Subsequently, a ‘well-fixed’ Exeter CUMARS construct was performed (Figure 7). We mixed 4 g of Vancomycin powder and 320 mg, equivalent to 8 ml of Gentamicin liquid, into each cement pack (40 g). A total of three cement packets were used (one acetabulum and two femurs). Tissue samples from the hip and acetabulum grew Enterococcus faecalis on cultures, which was consistent with our hip aspiration finding.

Postoperatively, she was started on 2 g of IV ampicillin every four hours for two weeks, followed by 2 g of oral ampicillin every eight hours for 10 weeks. She was allowed immediate full-weight bearing as tolerated with the aid of a walking frame, which she used for the first two weeks; thereafter, she was fully weight-bearing independently. Her neuropathic pain resolved immediately after surgery.

**Figure 8:**
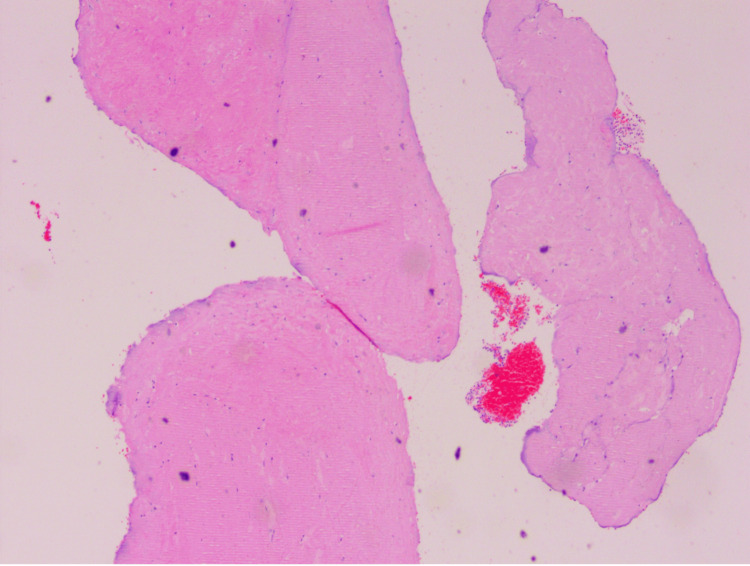
Histopathological microscopic examination showing numerous ovoid, biconvex to irregularly shaped amorphous eosinophilic configurations, admixed with occasional bits of inflammatory tissue and exudates

Microscopic examination of the rice bodies showed numerous ovoid, biconvex to irregularly shaped amorphous eosinophilic configurations, admixed with occasional bits of inflammatory tissue and exudates (Figure 8). There was no evidence of granulomas or malignancy.

Our patient is currently well and infection-free. She is able to ambulate freely with no further complaints.

## Discussion

Loose rice body formation organised in a cyst is a rare occurrence, and this is only the third reported case of rice-body cyst formation in patients with prior THA [5]. Furthermore, this is the first reported case of rice body formation associated with an infected THA.

It is widely believed that the formation of rice bodies is commonly associated with an underlying chronic inflammatory condition, such as rheumatoid arthritis and tuberculosis [11]. Of note, our patient did not have any of these underlying conditions. The pathophysiology of such a phenomenon is poorly understood. However, it is believed that the formation of these rice bodies is largely of synovial membrane origin, formed due to the shedding of the synovial membrane, which occurs secondary to synovial infarction [12]. Regardless of the underlying aetiology, synovial inflammation plays a distinctive role in the pathogenesis of rice bodies.

The aims of treatment for our patient include the eradication of infection and complete excision of the rice body cyst. Excision of the cyst is essential to alleviating its compressive effect on surrounding neurovascular structures. In our patient, this cyst containing rice bodies was compressing her sciatic nerve, resulting in neuropathic pain. It resolved spontaneously after her revision of the THA surgery.

While the complete excision of the cyst is essential, the underlying cause of synovial inflammation must be addressed. In our case, revision surgery utilising a ‘well-fixed’ Exeter CUMARS construct was deemed suitable for this case as her Enterococcus faecalis was sensitive to Vancomycin, which is a heat-stable antibiotic.

The Exeter Hip Unit in the United Kingdom pioneered using the CUMARS construct, which initially integrated a femoral stem on a polyethylene cup, along with antibiotic-loaded bone cement, which was ‘loosely fixed’ [13]. Many improvements have been made since then, and the CUMARS construct can now be retained indefinitely to avoid the costs, morbidity, and burden of a second-stage revision [13]. Another essential part of the management includes the use of antibiotic-loaded cement spacers, with the antibiotic of choice tailored to pre-operative hip aspiration culture results [13].

For our CUMARS construct, in addition to mixing 4 g of antibiotics in powder form depending on antibiotic sensitivity per mix of 40 g of bone cement, we also routinely mix 320 mg of gentamicin (8 ml). The addition of this liquid form of gentamicin is miscible, and it gives the bone cement a better consistency for cementation, as adding only 20 ml of liquid monomer to 40 g of bone cement with the addition of 4 g of antibiotic powder will yield a powdery consistency, which is poor for bone interdigitation. Studies have shown that increased bone cement porosity and the release of vancomycin are strongly enhanced by 146% with the addition of liquid gentamicin [14]. The mechanical property of bone cement is reduced with the addition of liquid gentamicin, but this is clinically not relevant in the context of a cement spacer [14].

## Conclusions

While there are increasing reports of rice body formation complicating hip arthroplasties in the literature, this phenomenon remains poorly studied. Identification of rice body cysts is essential, as they can be definitively managed with excision and sent for further histopathological analysis to rule out other pathologies. This ensures timely relief of symptoms, prevents further compression on neurovascular structures, and ultimately addresses the patient’s concerns and improves the patient’s quality of life.
